# The evolution of the concept of stress and the framework of the stress system

**DOI:** 10.15698/cst2021.06.250

**Published:** 2021-04-26

**Authors:** Siyu Lu, Fang Wei, Guolin Li

**Affiliations:** 1 Center for Aging Biomedicine, Key Laboratory of Protein Chemistry and Developmental Biology of Fish of Ministry of Education, College of Life Sciences, Hunan Normal University, Changsha, Hunan 410081, China.; 2 National & Local Joint Engineering Laboratory of Animal Peptide Drug Development, College of Life Sciences, Hunan Normal University, Changsha, Hunan 410081, China.; 3 Key Laboratory of Hunan Province for Model Animal and Stem Cell Biology, School of Medicine, Hunan Normal University, Changsha, Hunan 410081, China.

**Keywords:** stress, homeostasis, concept, definition, eustress, distress, sustress

## Abstract

Stress is a central concept in biology and has now been widely used in psychological, physiological, social, and even environmental fields. However, the concept of stress was cross-utilized to refer to different elements of the stress system including stressful stimulus, stressor, stress response, and stress effect. Here, we summarized the evolution of the concept of stress and the framework of the stress system. We find although the concept of stress is developed from Selye's “general adaptation syndrome”, it has now expanded and evolved significantly. Stress is now defined as a state of homeostasis being challenged, including both system stress and local stress. A specific stressor may potentially bring about specific local stress, while the intensity of stress beyond a threshold may commonly activate the hypothalamic-pituitary-adrenal axis and result in a systematic stress response. The framework of the stress system indicates that stress includes three types: sustress (inadequate stress), eustress (good stress), and distress (bad stress). Both sustress and distress might impair normal physiological functions and even lead to pathological conditions, while eustress might benefit health through hormesis-induced optimization of homeostasis. Therefore, an optimal stress level is essential for building biological shields to guarantee normal life processes.

## INTRODUCTION

Over the past decades, the concepts of stress have evolved and expanded significantly. Although the current concept of stress is developed from the pioneering contributions of Claude Bernard [1], Walter B. Cannon [2], and Hans Selye [3], stress no longer specifically refers to the acute activation of hypothalamic-pituitary-adrenal (HPA) axis and a series of compensatory sympathoadrenal responses when homeostasis is threatened [2, 3]. It is now clear that even lower organisms or isolated tissues and cells also have stress responses [4, 5]. Meanwhile, with the specifying of the concept of homeostasis, the concept of stress is becoming more and more specific. For instance, oxidative stress specifically refers to a disruption of redox signaling and control [6, 7], and endoplasmic reticulum stress refers to the stress induced by the accumulation of unfolded proteins in the endoplasmic reticulum [8]. With the extension of the concept of homeostasis, the concept of stress has permeated our culture in many aspects, it has become a core concept in the field of biology and medicine, and has been widely used in psychological, physiological, social, and environmental fields.

The implications of stress have expanded greatly. It now includes not only the negative aspects such as “general adaptation syndrome (GAS)” defined by Selye, threats to health and life, but also the positive aspects such as adapting to the existing environment and anticipating future challenges. Therefore, even Selye also suggests that it should be better to categorize stress into “eustress” meaning good stress, and “distress” meaning bad stress [9]. However, many scientists still use Selye's “GAS” to define stress, and simply interpret stress as a threat to health. An important reason for this is that the concept of stress itself has not been uniform. As Selye said, the concept of stress is often confused with the concept of stressful stimulus, stressor, stress response, and stress effect [9].

Therefore, this paper will first construct the basic framework of the stress system, and then summarize the key developments that have contributed to shaping the framework. Through this way, we hope the mature framework of the stress system will help to integrate stress-related concepts from disparate fields of science and medicine, and allow the concept of stress to be common across different fields.

## THE BASIC FRAMEWORK OF THE STRESS SYSTEM

**Figure 1:**
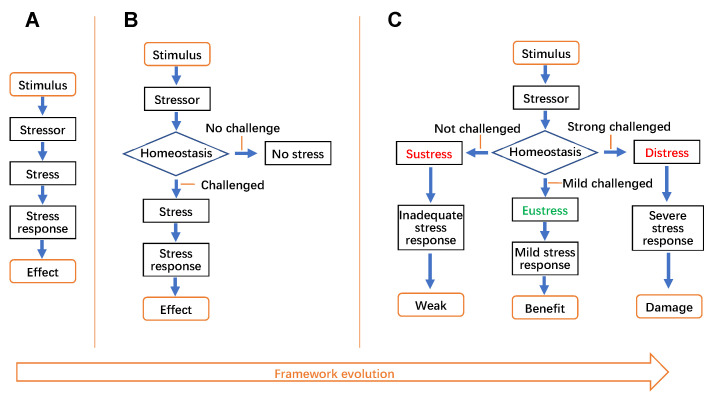
FIGURE 1: The evolution of the framework of the stress system. **(A)** The basic framework of the stress system. **(B)** The developing framework of the stress system. **(C)** The mature framework of the stress system.

Many scientists have noticed that the stress system contains several elements, such as stressful stimulus, stressor, and stress response [4, 5, 9]. Considering that the process of stress acting on the body is generally similar to other signal transduction processes, it should also include stimuli, receptors, and cascades. Therefore, we suggest the framework of the stress system should comprise five basic elements: stressful stimulus, stressor, stress, stress response, and stress effect (**Figure 1A**). In this framework, the stressful stimulus is the starting point, the effect is the end point, and stressor, stress, and stress response are cascades.

According to this framework, we may easily differentiate each element of the stress system. Taking oxidative stress as an example, reactive oxygen species (ROS) are stressors, the factors that stimulate the generation of ROS are stressful stimuli, a disruption of redox signaling and control caused by ROS is oxidative stress, the response that the body attempts to restore redox homeostasis is an oxidative stress response, and the resulting biological consequence is the effect of this stress.

Although the concept of stress is still confusing and controversial in many scientific pieces of literature, this framework may help readers to know the real meaning of stress in literatures. For instance, Selye defined “GAS” [3] is, in fact, both stress response and stress effect: the activation of the HPA axis is a systematic stress response, while physical and mental disorders produced by prolonged stress are stress effects. All treatments he used including heat, cold, and other noxious agents, are stressful stimuli. Since this paper has not revealed whether these stimuli are directly transferred into specific stressors, or induce the generation of specific stressors, it cannot be defined in which stress they have originally resulted in. But as Selye noticed, all these treatments caused a similar HPA activation-related stress response. To explain this phenomenon, Selye suggests that the stress is nonspecific and shared response, regardless of the nature of causative agents, or stressors [3, 9]. Later, this view of nonspecific stress response has been widely challenged [4, 10, 11]. This nonspecific stress response is not universal, especially it does not exist in isolated cells and tissues [4, 10]. However, as the HPA axis is an “alarm system” for higher animals, in a sense, all stresses, once their intensity exceeds a certain threshold, may potentially cause HPA axis-related systematic stress response [5, 12].

## THE CONCEPTS OF STRESS

Notably, the above basic framework of the stress system still has no “sensor”, thus it is difficult to determine which stressors may result in stress and which may not. Fortunately, in the 1920s, Cannon coined “homeostasis [13]” referring to the tendency of a system to maintain the stability of milieu intérieur [1], and found a wide variety of threats to homeostasis causing a similar sympathoadrenal response that he termed “fight-or-flight” response [14, 15], which we now know is a typical stress response. Therefore, homeostasis might be the candidate “sensor” of the stress system. Cannon defined stress as threats to homeostasis [2]. Selye also found the activation of HPA axis was a common response to diverse nocuous agents or sublethal doses of intoxications, and defined stress as the “nonspecific response of the body to any demand upon it” [3, 16, 17].

Obviously, according to the above framework, stress defined by Cannon is stressor, and Selye's stress and Cannon's “fight-or-flight” response are stress responses. However, through introducing homeostasis into the concept of stress, their works promote the evolution of the stress system, as homeostasis endows the framework of the stress system with the ability to sense stressors and judge whether they are threats or not (**Figure 1B**). With the help of homeostasis, it is easy to understand that not all stressors inevitably cause stress, but the stressors that threaten homeostasis do [5].

It is now clear that stress should be a state rather than a stressor or response [5], which has also been mentioned by Selye [9]. Based on homeostasis, all elements of the stress system can be clearly defined (**BOX 1**). Stressors are factors with the potential to directly challenge homeostasis. Stress is a state of homeostasis being challenged. Stressful stimuli are agents that can induce the formation of stressors or transfer to stressors. Stress response is a compensatory process aimed to restore homeostasis. Stress effects are biological consequences resulting from the struggle with stressors, which may include re-establishing homeostasis that promotes health (positive effects), or causing damage to the body or even diseases (negative effects).

**Box 1:**
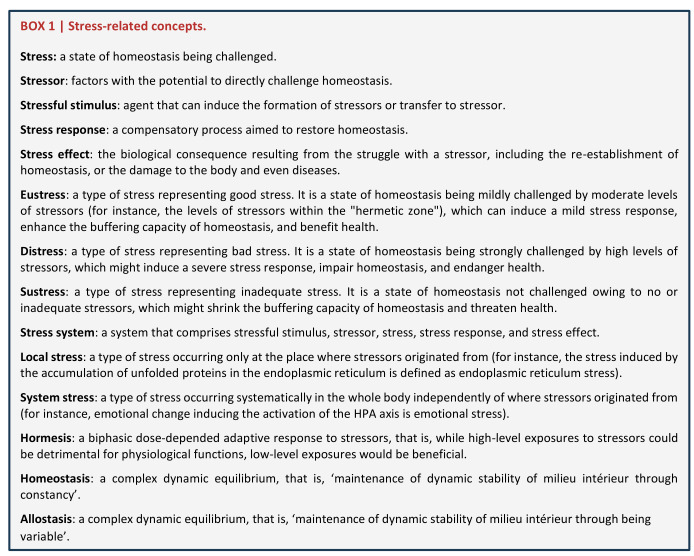
Box 1 Stress-related concepts

## EXTENSION AND SPECIFICITY OF THE CONCEPT OF STRESS

Initially, the term homeostasis coined by Cannon was a purely physiological concept in animals. Later, the concept was extended to the field of psychology, and cumulating evidence suggested that the activation of the HPA axis was more sensitive to emotional activities than physiological ones (**Table 1**). Therefore, stress was categorized into physiological stress and psychological stress [10]. Subsequent studies revealed that although different psychological activities could commonly activate the HPA axis, the phenotypes and mechanisms of corresponding stress were different from each other. Therefore, psychological stress was further classified into four main types according to specific functions (**Table 2**), that is, emotional stress [18], cognitive stress [19], perceptual stress [20], and psychosocial stress [21], and each type was sometimes further classified according to specific psychological stressors or stimuli, such as social defeat stress [21, 22], post-traumatic stress [23–25], and pandemic stress [26, 27].

**TABLE 1.** Summary of stress responsive system.

**Organisms**	**Stresses**	**Stress-responsive system**	**Functions/Effects**	**Typic stress or syndrome**	**Refs**
**Fungi**	**Environmental stress**	Antioxidant defense system	Adaptation, growth inhibition, *et al.*	Oxidative stress	[47]
**Plants**	**Abiotic stress**	Transcription factors, antioxidant defense system, *et al.*	Adaptation, cell death, oxidative damage, *et al.*	Heat stress	[33, 67, 68]
**Animals**	**Physiological stress**	Hormones, transcription factors, antioxidant defense system, *et al.*	Hormesis, structural damage, functional loss, *et al.*	ER stress	[69–71]
	**Psychological stress**	Hormones, HPA axis, *et al.*	Depression, *et al.*	Post-traumatic stress disorders	[72, 73]

Unlike psychological stress, which is mainly system stress, physiological stress includes both system stress and local stress. Therefore, only the specificity of systematic physiological stress is similar to that of psychological stress, that is, termed by specific stimuli or stressors (**Table 2**), such as oxidative stress [28], nutrient stress [29], heat stress [30], thermal stress [31], shear stress [32], drought stress [33], osmotic stress [34], mechanical stress [35], genotoxic stress [36], and so on. However, the specificity of most local physiological stress is termed according to the sites where stressors are produced (**Table 2**). For instance, cardiac stress [37], dopamine neuron-specific stress [38], cytoskeletal stress [39], mitochondrial stress [40, 41], endoplasmic reticulum stress [8, 42], and telomere stress [43] are stresses taking place only in specific cellular or subcellular sites. Besides, some physiological stresses are also classified by functions (**Table 2**), such as metabolic stress [44], replication stress [45], and neurodegenerative stress [46].

**TABLE 2.** The summary of stress types.

**Organisms**	**Stresses**			**Stimuli**		**Refs**
	**Types**	**Subtype Basis**	**Subtype examples**	**Type**	**Examples**	
**Fungi**	**Environmental stress**					[47]
	Stimuli		Oxidative stress	Chemical	H_2_O_2_ Menadione Sodium bisulphite	
			Osmotic stress		NaCl Sorbitol	
			CWI stress		Congo Red	
			Heavy metal stress		CdCl_2_	
			*et al.*		*et al.*	
**Plants**	**Abiotic stress**					
	Stimuli		Drought stress Heat stress Salt stress *et al.*	Physical	Drought Temperature Salt *et al.*	[74–76] [67, 77] [75]
			Oxidative stress *et al.*	Chemical	ROS Toxic chemicals	[48, 68, 78] [79]
					*et al.*	
**Animals**	**Physiological stress**					
	Stimuli		Cold stress Heat stress Radiation stress Noise stress Vibration stress *et al.*	Physical	Cold Heat Radiation Noise Vibration *et al.*	[80, 81] [82, 83] [84] [85, 86] [87]
			Chemical stress *et al.*	Chemical	Chemicals	[88]
			Chemical stress	Biological	Metabolites	[89]
			*et al.*		*et al*	
	Functions		Metabolic stress			[44]
			Replication stress			[45]
			Neurodegenerative stress			[46]
			*et al.*			
	Locations		Cardiac stress			[37]
			Dopamine neuron-specific stress			[38]
			Cytoskeletal stress			[39]
			Mitochondrial stress			[40, 41]
			Endoplasmic reticulum stress			[8, 42]
			Telomere stress			[43]
			*et al.*			
	Functions		Emotional Stress	Emotional	Anxiety Fear Grief Anger *et al.*	[90] [91, 92] [92] [92]
			Cognitive stress	Cognitive	Pandemic Information overload Disruptions Interruptions Aging *et al.*	[27] [93] [93] [93] [19]
			Perceptual stress	Perceptual	Aging Addiction Competition *et al.*	[94, 95] [96] [97]
			Psychosocial stress	Psychosocial	Social defeat Social confrontations Racial disparity Trauma from life events *et al.*	[21, 22] [22] [98] [25]

Notably, the concept of stress was also used in filamentous fungi [47] and plants [48]. Although these stresses mainly belonged to physiological stress, they were specifically termed as environmental stress and abiotic stress (**Table 2**), respectively. The subtypes of both environmental stress and abiotic stress were classified mainly according to stimuli (**Table 2**).

Besides, owing to the implications of homeostasis have extended to social and environmental science, the concept of stress has also expanded to related fields. Therefore, some specific stress concepts in social and environmental science have also been coined by related scientists [26, 47, 49, 50].

## STRESS, HORMESIS, SUSTRESS, EUSTRESS, DISTRESS, HOMEOSTASIS AND ALLOSTASIS

For many scientists, the word stress is still closely associated with Selye defined “GAS” [3], and it seems all stress responses are negative for health. But over the last 80 years, science has expanded the concept of stress along many dimensions. Particularly, accumulating evidence illustrates that most stressors display a biphasic dose-dependent effect on health, that is, while high-level exposures to stressors could be detrimental to health, low-level exposures would be beneficial [51]. These biphasic dose responses have been defined as “hormesis” (**BOX 1**) [52], and the low dose adaptive response is considered to be the result of compensatory biological processes to restore homeostasis perturbed by stressors [52]. A typical example is ROS-induced oxidative stress [53–56]. ROS are reactive molecules with the potential to damage proteins, lipids, nucleic acids, and other biomolecules [55]. Early studies suggested that ROS contributed to the pathogenesis of many diseases, and even promoted the aging process [57]. However, it is now clear that although excessive ROS may cause damage to biomolecules, maintenance of an optimal level of ROS is essential for modulating life processes [28, 55, 58, 59]. Therefore, more and more researchers have come to realize that an optimal stress level is crucial for health, while an excessive or inadequate stress level might impair development, growth, and body composition, and lead to pathological conditions [5]. In this context, stress has to be divided into eustress, distress, and sustress (**Figure 1C**, **BOX 1**). Here, eustress means good stress, that is, homeostasis has been mildly challenged by moderate levels of stressors (for instance, the levels of stressors within the “hermetic zone” [60]). Eustress might induce a mild stress response, enhance the buffering capacity of homeostasis [61], and benefit health. Distress means bad stress, that is, homeostasis has been strongly challenged by high levels of stressors, which might induce a severe stress response, impair homeostasis, and endanger health. Sustress is coined from the Latin ‘sus' (an assimilated form of the Latin “sub-” before “-s-”) meaning ‘less than normal' and ‘stress' to mean ‘no or inadequate stress'. Sustress might shrink the buffering capacity of homeostasis [61] and threaten health [5, 58]. The above “GAS” [3] only represents the responses and effects resulting from distress, but not eustress and sustress.

**Figure 2:**
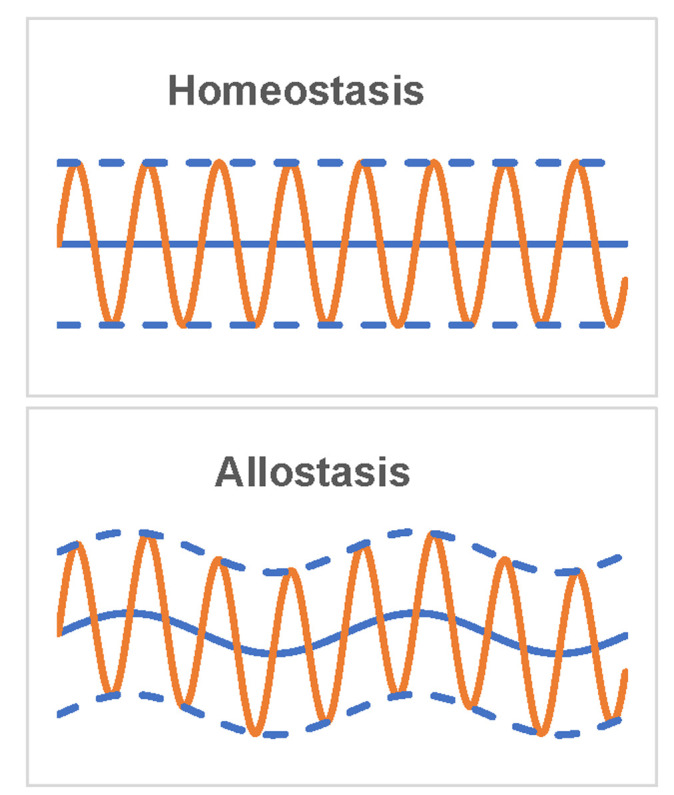
FIGURE 2: Homeostasis and allostasis. Blue solid line represents the baseline, orange line represents dynamic equilibrium, and blue dot line represents the boundary of dynamic equilibrium.

In this framework of the stress system (**Figure 1C**), homeostasis likes a ‘commander', which senses different stressors and directs subsequent stress responses and effects. Notably, although the concept of homeostasis has still been widely used, its original meaning of ‘maintenance of dynamic stability of milieu intérieur through constancy' (**Figure 2**) has been challenged, because the baseline of homeostasis might be dynamic rather than constant. Accumulating evidence indicates that many physiological indexes and activities including blood pressure, body temperature, the secretion of hormones, and the expression of proteins and genes, display a typical circadian rhythm. Therefore, in 1988, Sterling and Eyer coined a new term ‘allostasis' (**BOX 1**) from the Greek ‘allo' meaning ‘variable', and ‘stasis' meaning ‘stable', to represent ‘‘remaining stable by being variable' [62]. As illustrated in **Figure 2**, the only difference between these two concepts is that the baseline of allostasis is variable, while that of homeostasis is constant. Most of the dynamic equilibrium of milieu intérieur should be allostasis rather than homeostasis. In this context, McEwen coined the term “allostatic load” to describe the stress process [63, 64].

## SUMMARY AND PERSPECTIVES

Stress is a state of homeostasis being challenged. Along with the concept of homeostasis extending to the fields of physiology, psychology, and even environmental science, the concept of stress has evolved dramatically. It is now clear that stress might take place systematically through activating the HPA axis (system stress) or take place only at the site where stressors are induced or generated (local stress). Since any stressor may be sensed by existing homeostasis and potentially trigger responses at molecular, cellular, and systemic levels to preserve the homeostasis and induce adaptation, the concept of stress has been specified according to different stressful stimuli, stressors, sites, or functions, such as emotional stress, oxidative stress, mitochondrial stress, metabolic stress, and so on. As research continues, many more specific stresses will emerge.

Stress functions through the stress system, and it contains three basic types: distress, eustress, and sustress. While distress may impair normal physiological function, eustress plays a critical role in the adaptive process of assessing and disposing of stressors, and endowing the individual to prepare for and survive future challenges [65], and sustress may weaken the basal activity and responsiveness of the stress system [5]. Therefore, more and more researchers have come to realize that an optimal stress level is essential for building biological shields through hormesis to guarantee normal life processes [5, 66].

The word of stress has permeated our culture in many dimensions, while the stress concept is still confusing and controversial. We hope this framework of the stress system will help distinguish the true meaning of the stress concept appearing in different pieces of literature, integrate stress-related concepts from disparate fields of science and medicine, and allow the concept of stress to be common across different fields.

## AUTHOR CONTRIBUTIONS

G.L. and S.L. wrote the manuscript, and F.W. and G.L. revised the manuscript.

## Abbreviatons:

GAS: – general adaption syndrome,
HPA: – hypothalamic-pituitary-adrenal,
ROS: – reactive oxygen species.
